# Adaptive Feature Medical Segmentation Network: an adaptable deep learning paradigm for high-performance 3D brain lesion segmentation in medical imaging

**DOI:** 10.3389/fnins.2024.1363930

**Published:** 2024-04-12

**Authors:** Asim Zaman, Haseeb Hassan, Xueqiang Zeng, Rashid Khan, Jiaxi Lu, Huihui Yang, Xiaoqiang Miao, Anbo Cao, Yingjian Yang, Bingding Huang, Yingwei Guo, Yan Kang

**Affiliations:** ^1^School of Biomedical Engineering, Shenzhen University Medical School, Shenzhen University, Shenzhen, China; ^2^College of Health Science and Environmental Engineering, Shenzhen Technology University, Shenzhen, China; ^3^School of Applied Technology, Shenzhen University, Shenzhen, China; ^4^Guangdong Key Laboratory for Biomedical Measurements and Ultrasound Imaging, School of Biomedical Engineering, Medical School, Shenzhen University, Shenzhen, China; ^5^College of Big Data and Internet, Shenzhen Technology University, Shenzhen, China; ^6^College of Medicine and Biological Information Engineering, Northeastern University, Shenyang, China; ^7^Shenzhen Lanmage Medical Technology Co., Ltd, Shenzhen, China; ^8^School of Electrical and Information Engineering, Northeast Petroleum University, Daqing, China

**Keywords:** medical image analysis, brain lesion segmentation, adaptive feature extraction, attention mechanism, encoder-decoder architecture, computer-aided diagnosis, deep learning, neurological diagnostics

## Abstract

**Introduction:**

In neurological diagnostics, accurate detection and segmentation of brain lesions is crucial. Identifying these lesions is challenging due to its complex morphology, especially when using traditional methods. Conventional methods are either computationally demanding with a marginal impact/enhancement or sacrifice fine details for computational efficiency. Therefore, balancing performance and precision in compute-intensive medical imaging remains a hot research topic.

**Methods:**

We introduce a novel encoder-decoder network architecture named the Adaptive Feature Medical Segmentation Network (AFMS-Net) with two encoder variants: the Single Adaptive Encoder Block (SAEB) and the Dual Adaptive Encoder Block (DAEB). A squeeze-and-excite mechanism is employed in SAEB to identify significant data while disregarding peripheral details. This approach is best suited for scenarios requiring quick and efficient segmentation, with an emphasis on identifying key lesion areas. In contrast, the DAEB utilizes an advanced channel spatial attention strategy for fine-grained delineation and multiple-class classifications. Additionally, both architectures incorporate a Segmentation Path (SegPath) module between the encoder and decoder, refining segmentation, enhancing feature extraction, and improving model performance and stability.

**Results:**

AFMS-Net demonstrates exceptional performance across several notable datasets, including BRATs 2021, ATLAS 2021, and ISLES 2022. Its design aims to construct a lightweight architecture capable of handling complex segmentation challenges with high precision.

**Discussion:**

The proposed AFMS-Net addresses the critical balance issue between performance and computational efficiency in the segmentation of brain lesions. By introducing two tailored encoder variants, the network adapts to varying requirements of speed and feature. This approach not only advances the state-of-the-art in lesion segmentation but also provides a scalable framework for future research in medical image processing.

## 1 Introduction

Artificial intelligence (AI) in medical imaging has led to a new era in the healthcare system (Hassan et al., 2022). AI-based medical imaging diagnosis facilitates doctors to detect abnormalities earlier, allowing for early control of diseases (Yang and Yu, 2021). One example is the various imaging modalities, such as magnetic resonance imaging (MRI), computed tomography (CT), and ultrasound machines, which enable detailed visualization of structures within the body (Hurlock et al., 2009). To fully utilize these abilities, the detailed medical image segmentation (MIS) process requires careful marking of organs and lesions, slice by slice. This step is essential in radiology, particularly for identifying and monitoring disease conditions. It is a big challenge due to the varied nature of brain lesions and stroke data, the complex structure of the brain itself, as well as significant amounts of MRI and CT scans (Siuly and Zhang, 2016). The precision of segmentation has an impact on diagnosing, treating, and combating nervous system disorders, which account for many deaths around the world (Stoyanov et al., 2018). In recent years, Deep Learning (DL) techniques have greatly simplified medical segmentation. Consequently, there is more research into automating brain lesion detection and segmentation (Wang et al., 2022; Ma et al., 2023). Because of such technological progress, manual and semi-manual are greatly improved. These improved experiences resulted in earlier interventions and better patient results.

Advances in DL approaches have greatly improved the segmentation of medical images, providing significant performance and adaptability to different medical image applications (Greenspan et al., 2023). However, using these methodologies can also pose several challenges. Due to most DL networks’ intricate layers and parameters, training takes a long processing time and computational cost. Additionally, consider applying these approaches in a specific imaging situation, such as a brain lesion with split pixel imbalances and complex structures. The segmentation process becomes more complex and less efficient (Shatnawi et al., 2018). Considering these minor errors can significantly affect the performance of these techniques, designing and configuring them for specific problems needs a high level of expertise (Li et al., 2020). Image modalities, imagine size, voxel spacing, and class ratio can all have a substantial impact on 3D medical imaging performance (Vedaei et al., 2023). In addition, to effectively use these approaches, memory requirements, processing capability, and task-specific expertise must be addressed (Celaya et al., 2022).

To address these issues in 3D medical images, we propose the Adaptive Feature Medical Segmentation Network (AFMS-Net). AFMS-Net consists of two encoder modules: Single Adaptive Encoder Block (SAEB) and Dual Adaptive Encoder Block (DAEB). Both versions aim to improve feature extraction and model interpretation. SAEB uses a squeeze-and-excite technique to improve feature representation while reducing model parameters. It is ideal for initial screenings and applications where computational efficiency is a priority. Conversely, DAEB integrates advanced attention mechanisms to capture local and global features, resulting in a comprehensive and precise representation of feature information. The DAEB is designed to address multi-class segmentation challenges datasets such as BRATS, where accurate segmentation with fine-grained and multiple-class labels is essential. This module is particularly useful in cases involving multi-class lesions, where the size, shape, and location of each lesion may significantly influence the diagnosis and treatment plan. Then, incorporate a novel SegPath module between the encoder and the decoder to eliminate the semantic gap and boost feature refinement. The AFMS-decoder utilizes simple convolutional layers and transpose layers to illustrate the respective encoder’s features. The proposed AFMS-Net strikes the stability between computational efficiency and segmentation performance, demonstrating impressive findings across three diverse medical datasets in single and multiclass segmentation tasks. Therefore, the suggested segmentation framework shows a significant benchmark for future research in medical image diagnosis.

The key contributions of our research are summarized as follows:

1. We designed an encoder-decoder framework called the Adaptive Feature Medical Segmentation Network (AFMS-Net) framework for brain lesion segmentation.
2. We propose two different encoder modules, a Single Adaptive Encoder Block (SAEB) and a Dual Adaptive Encoder Block (DAEB). SAEB, designed for efficiency, employs a Squeeze-and-Excitation mechanism to capture sufficient primary features from the input images. In contrast, DAEB, is embedded in our AFMS-Net targeting complex cases like BRATS, uses a detailed attention mechanism that considers advanced channel-wise and spatial data.
3. The strategic placement of the new SegPath between the network’s encoder-decoder modules addresses the problem of gradient vanishing, boosting feature refinement, and aggregation for enhanced segmentation features. The introduction of an AFMS decoder illustrates the respective encoder’s features.
4. Comprehensive experimental analysis was conducted across three standard MIS datasets (BraTS, ALTAS, and ISLES), and seven different state-of-the-art approaches were compared. Our findings show that the AFMS-Net’s robust performance and generalization capability across different datasets emphasize its potential as a new benchmark for segmenting medical images based on standard evaluation metrics.

## 2 Related work

### 2.1 Brain lesion segmentation

There has been significant progress in brain lesion segmentation and advanced imaging techniques in recent years. However, accurate segmentation still poses a challenge. Traditional approaches mainly incorporate model-driven techniques, which rely on handcrafted features such as intensity distributions, gradients, morphological attributes, and texture characteristics. Using a voxel probability estimation approach, Anbeek et al. (2004) segmented white matter lesions from brain MRI images. Furthermore, Gooya et al. (2012) combined multi-channel MRI with probabilistic models to show the adaptability of conventional techniques. Moreover, Islam et al. (2013) presented an advanced method of brain tumor segmentation based on spatial and intensity characteristics.

Recently, deep learning has made a significant contribution to brain lesion segmentation. Numerous automated techniques have been proposed, including fully-supervised, supervised unsupervised, and atlas-based methods. So far, convolutional neural network (CNN) based deep learning techniques have demonstrated exceptional performance in medical imaging. The U-Net (Ronneberger et al., 2015) model’s efficient encoder-decoder structure has become a starting point for many advanced medical segmentation methodologies. Çiçek et al. (2016) expanded the U-Net architecture into 3D to handle the volumetric data. Based on U-Net, Zhou et al. (2018) developed nested U-Net (Unet++), which minimizes the loss of semantic information between the encoder and decoder. During the 2018 BRATS challenge, Myronenko (2019) proposed a densely connected convolutional blocks auto-encoder model for enhanced brain tumor segmentation. Huang et al. (2020) introduced a full-scale skip connection method through the integration of high-resolution and low-resolution data at various scales. In the Double U-Net network (Guo et al., 2021), two U-Net networks are sequentially organized, in which an Atrous Spatial Pyramid Pooling (ASPP) is placed after every down sample layer in the encoder. In the evaluation, Double U-Net segments nuclei and lesion boundaries well. A gradient vanishing problem has been observed during the converging process of deeper networks. To overcome this problem, Limonova et al. (2021) developed the ResNet-like architecture model. As a contribution to this growing research, Isensee et al. (2021) developed nnU-Net, a self-configuring method for medical image segmentation that adapts based on the provided dataset. According to Rashid et al. (2021) deep learning can automatically segment cerebral microbleeds from structural brain MRI scans. Furthermore, Kermi et al. (2022) developed a multi-view CNN combining the advantages of 2D and 3D networks for glioma segmentation. These findings highlight the various and constantly developing uses of deep learning for medical image segmentation. This research aims to gradually increase segmentation performance, boost efficiency, and address specific issues related to lesion patterns across various illnesses.

Despite all of the advancements made, some issues still need to be resolved in this field. Precisely identifying lesion boundaries remains a challenge for appropriate diagnosis and treatment planning. Secondly, the class imbalance issue often leads to suboptimal model performance in medical imaging datasets, where lesions are considerably smaller than the non-lesion areas. In addition, multi-class lesions, where a single brain scan might reveal several different types of lesions that must be segmented concurrently, remain an open issue. The aim should be to overcome these challenges to design more accurate, effective, and reliable techniques for brain lesion segmentation. The proposed framework addresses these issues using an advanced attention-based deep-learning approach.

### 2.2 3D attention mechanism in medical imaging data

Attention mechanisms recently gained popularity in computer vision, particularly in medical image segmentation (Gao et al., 2023). This technique, which is well-known for its precise feature selection, enhances the effectiveness of CNNs for a wide range of complex tasks, including detection and classification problems. Squeeze-and-Excitation Network (SENet) (Hu et al., 2018) is a well-illustrated example of an attention mechanism. In SENet, Squeeze-and-Excitation modules determine how feature map channels interact to gather global spatial information. Inspired by SENet, Oktay et al. (2018) designed attention U-Net architecture. This approach reduced the need for extra computational resources or model parameters by accurately targeting regions and highlighting valuable features using a novel bottom-up attention gate. As the field progressed, more sophisticated models began to emerge. Wang et al. (2019) introduced the Volumetric Attention (VA) mechanism, capable of creating 3D enhanced attention maps across spatial and channel dimensions, specifically targeting areas of interest like liver tumors in CT scans. Taking a different approach, Zhang et al. (2020) developed employing attention guidance to enhance segmentation decoders’ ability to perceive 3D contexts. Mou et al. (2021) proposed self-attention mechanism, particularly effective in segmenting curved structures such as nerves and blood vessels. This proposal opened new avenues for future research and advancements in the field. In the most recent developments, Zeng et al. (2023) introduced the Multi-Scale Reverse Attention modules (MSRAM) to capture fine-grained features in 3D brain vessel images at different scales. Several promising methods (Nie et al., 2022; Mehrani and Tsotsos, 2023) have developed due to the advancement of attention mechanisms in 3D medical image segmentation. As the field progresses, we optimize existing attention architectures and propose a lightweight, enhanced attention-based model to segment 3D medical images precisely.

## 3 Methodology

### 3.1 Overall architecture

**Figure 1:**
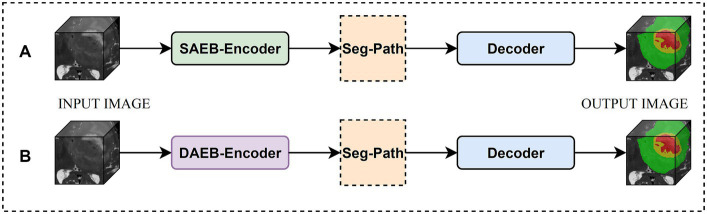
Overview of the AFMS-Net. **(A)** Encoder-decoder with SAEB. **(B)** Encoder-decoder with DAEB.

**Figure 2:**
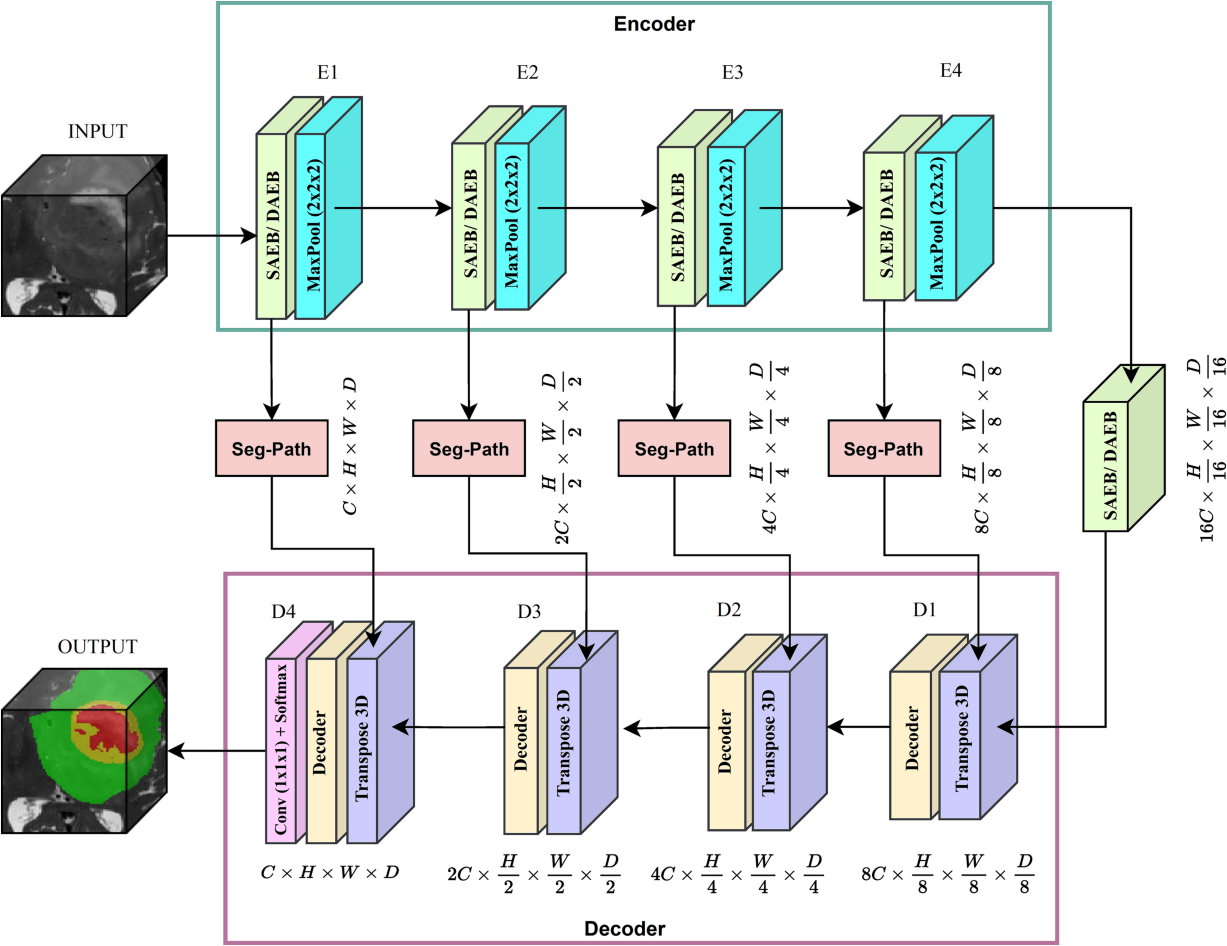
Proposed brain lesion segmentation pipeline. Adaptive encoder, SegPath and decoder.

We introduced two versions of AFMS-Net for segmenting brain lesions using the proposed SAEB, DAEB, SegPath, and decoder, as demonstrated in Figure 1. Different encoders (SAEB/DAEB) are used in each version, allowing for capturing global and local feature information, enhancing the network’s representative ability and feature extraction process. Both versions follow the encoder-decoder structural design illustrated in Figure 2. The SAEB encoder block first uses the Squeeze-and-Excitation (SE) block to extract low-level features. It achieves this by recalibrating channel responses, thereby highlighting crucial details. Additionally, the fusion of 3 × 3 × 3 convolutional along with 1 × 1 × 1 convolutions serves to synthesize these features, further refining the high-level feature understanding. The DAEB module applies a dual-attention mechanism that emphasizes meaningful semantic features.

Initially, channel-wise attention is achieved through Global Average Pooling (GAP), reshaping, and convolutional layers. This approach enables the network to highlight features in specific channels selectively. The network then learns to focus on essential spatial regions by processing max-pooled and average-pooled information through a convolutional layer. Combining these two attention mechanisms results in a more focused and relevant feature map highlighting channel-specific and spatial information. Each SAEB and DAEB is followed by a 3 × 3 × 3 max pooling with stride 2 for a down-sampling operation. The SegPath module is strategically placed between the encoder and decoder, addressing gradient vanishing and increasing feature refining and aggregation for improved segmentation features. The AFMS-Net decoder gradually up samples the feature maps obtained by the encoder to correspond with the resolution of the input image. The final output of the AFMS-Net is a segmentation probability map obtained from a 3D convolutional layer followed by a softmax activation function, accurately identifying brain lesions. The distinguishing feature of AFMS-Net is its dynamic feature refinement, ensuring superior model results while maintaining computational efficiency. The two versions of the model allow us to evaluate and compare the efficiency and effectiveness of SAEB and DAEB in brain lesion segmentation. More detailed information about the components and operations of AFMS-Net are provided in the subsequent sections.

### 3.2 AFMS-Net encoder

#### 3.2.1 Single Adaptive Encoder Block

Medical image analysis presents unique challenges that require efficient and robust network architectures. While several network architectures like MobileNet (Howard et al., 2017), EfficientNet (Tan and Le, 2019), and PocketNet (Celaya et al., 2022) have contributed valuable approaches to handling complex features, they often grapple with a trade-off between performance and computational efficiency. Such as, Deeplabv3 (Yurtkulu et al., 2019) captures complex image features that demand significant computational resources. Deeplabv3 parallel convolutional pathways handle multi-scale features but at the cost of a complex architecture and high parameters count. MobileNet and EfficientNet introduced solutions used depth-wise separable convolutions and compound scaling. However, the goal for optimal efficiency and real-time processing continues.

**Figure 3:**
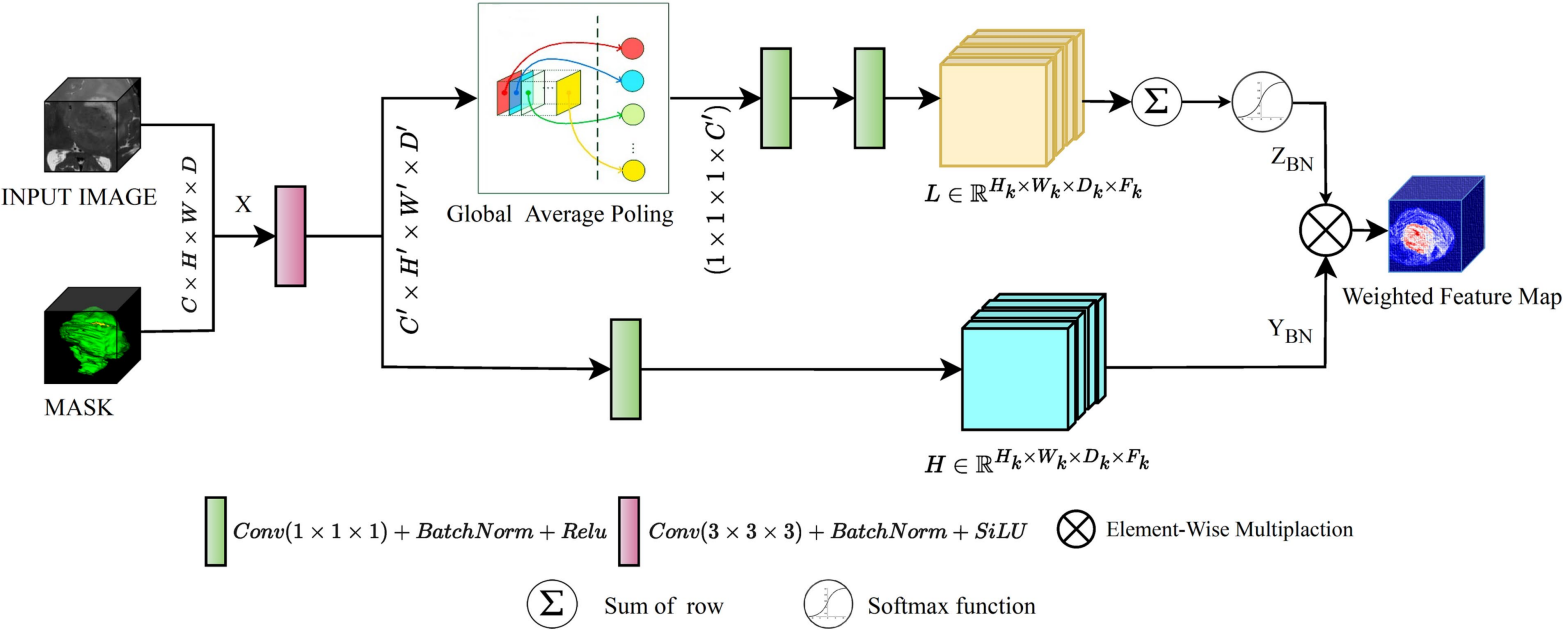
An illustration of the proposed SAEB module, the yellow rectangles representing low-level features and the blue rectangles representing high-level features.

In response to these challenges, proposed network balance computational efficiency with the capacity for effective feature extraction in medical image analysis. Inspired by the Squeeze-and-Excitation (SE) mechanism, SAEB begins the feature extraction process with a single 3D convolution layer. This approach initiates the feature extraction process with a single 3D convolution layer. An intermediate GAP operation follows, leading to the application of two 1×1×1 convolution layers. These layers act as channel-wise transformation agents within the SE mechanism, effectively managing dimensionality reduction and restoration. Figure 3 illustrates the transformations and operations performed within the SAEB, which are especially useful when interpreting complex patterns, such as segmenting brain lesions. The integration of 3×3×3 and 1×1×1 convolutions synthesizes and refines features, enhancing the model’s high-level feature understanding and representative ability.

For instance, we initiate this discussion with the examination of the 3D convolution layer which allows the model to handle the width, height, and depth dimensions of the input data, which is crucial in medical image analysis. Mathematically, the convolution operation involves an input tensor $X\in {\mathbb{R}}^{\left(H\times W\times D\times C\right)}$ and filter $F\in {\mathbb{R}}^{\left(H\times W\times D\times C\right)}$ , where each position $\left(i,j,k\right)$ in the output feature map $Y\in {\mathbb{R}}^{\left(H^{\prime }\times W^{\prime }\times D^{\prime }\times C^{\prime }\right)}$ is computed as follows.


(1) $$\begin{array}{l} Y\left(i,j,k,c^{\prime }\right)=\\ \;\overset{h-1}{\underset{a=0}{\sum }}\;\overset{w-1}{\underset{b=0}{\sum }}\;\overset{d-1}{\underset{c=0}{\sum }}\;\overset{C-1}{\underset{d=0}{\sum }}\;X\left(i+a,j+b,k+c,d\right)\times F\left(a,b,c,d,c^{\prime }\;\right) \end{array}$$

In Eq. 1, $Y\left(i,j,k,c^{\prime }\right)$ represents the value at the position $\left(i,j,k,c^{\prime }\right)$ in the output tensor Y . The four nested summations are indexed by variable a,b,c and d iterate over the ranges $\left[0,h-1\right]\left[0,w-1\right]\left[0,d-1\right]$ and $\left[0,C-1\right]$ respectively. These indices correspond to the spatial dimensions and channels of the input tensor X . The $X\left(i+a,j+b,k+c,d\right)$ represents the value at the position $\left(i+a,j+b,k+c,d\right)$ in the input tensor X and $F\left(a,b,c,d,c^{\prime }\right)$ describes the learnable parameters of the convolutional filter, where $c^{\prime }$ denotes the output channel index. The SAEB incorporates a Batch Normalization (BN) operation to ensure model stability and efficient training. BN normalizes the input feature maps, mitigating the issue of internal covariate shift and improving model stability and performance. The BN operation calculates the batch mean $E\left(Y\right)$ , variance $Var\left(Y\right)$ and utilizes learnable scale $\left(\gamma \right)$ and shift $\left(\beta \right)$ parameters to produce batch-normalized output $Y_{BN}$ . The following combined equation can represent the BN operation.


(2) $$Y_{BN}=\gamma \times \left(\frac{Y-E\left(Y\right)}{\sqrt{Var\left(Y\right)+\in }}\right)+\beta$$

In Eq. 2, initially, the batch mean $E\left(Y\right)$ is calculated as the mean of the input tensor across the mini-batch for each channel, ensuring the normalization process considers the distribution of inputs, as formalized in Eq. 3


(3) $$E\left(Y\right)=\frac{1}{m}\overset{m-1}{\underset{i=0}{\sum }}\;Y\left[i\right]$$

Following the computation of the $E\left(Y\right)$ , the batch variance $Var\left(Y\right)$ is calculated as the average of the squared differences between each element in the mini-batch and the batch mean, as described by Eq. 4.


(4) $$Var\left(Y\right)=\frac{1}{m}\overset{m-1}{\underset{i=0}{\sum }}\;{\left(Y\left[i\right]-E\left(Y\right)\right)}^{2}$$

Eq. 5, describes how the normalized output *ŷ* is obtained by subtracting the batch mean from the input tensor Y and dividing it by the square root of the batch variance plus a small constant ∈ for numerical stability.


(5) $$\hat{Y}=\gamma \times \left(\frac{Y-E\left(Y\right)}{\sqrt{Var\left(Y\right)+\in }}\right)$$

The final batch-normalized output $Y_{BN}$ is obtained by scaling the normalized output *ŷ* with the learnable scale $\left(\beta \right)$ and shift parameters as depicted in Eq. 6. This step customizes the normalization to the specifics of the data being processed.


(6) $$Y_{BN}=\left(\gamma \times \hat{Y}\right)+\beta$$

following the BN, the SAEB applies a GAP operation to the batch-normalized output $Y_{BN}$ as encapsulated in Eq. 7, which summarizes the presence of each feature across the spatial dimensions, resulting in a tensor $S\in {\mathbb{R}}^{\left(1\times 1\times 1\times c^{\prime }\right)}$ that captures the global information of the feature maps. The $\left(cth\right)$ element of S can be expressed as:


(7) $$\left[S_{c}\right]=\frac{1}{H^{\prime }\times W^{\prime }\times D^{\prime }}\overset{H^{\prime }-1}{\underset{i=0}{\sum }}\overset{W^{\prime }-1}{\underset{j=0}{\sum }}\overset{D^{\prime }-1}{\underset{k=0}{\sum }}\;Y_{BN}\left[i,j,k,c\right]$$

The GAP operation provides a global summary of each channel, capturing the overall presence of features across the spatial dimensions. Following the GAP operation, the SAEB applies a reshape operation to transform the GAP output into a suitable shape for subsequent operations. It is then passed through two 1 × 1 × 1 convolutions to perform channel-wise transformations. The first 1 × 1 × 1 convolution reduces the number of channels, while the second 1 × 1 × 1 convolution restores the original number of channels. The softmax activation operation is then applied to generate attention weights $A\left[c\right]$ that represent the importance assigned to each channel. This operation calculates a probability distribution across the channel dimension, yielding attention weights $A\left[c\right]$ given as follows:


(8) $$A\left[c\right]=\frac{\exp\left(S\left(c\right)\right)}{\sum_{d=0}^{C'-1}\exp\left(S\left(d\right)\right)}$$

In Eq. 8, $S\left(d\right)$ denotes the value of the GAP output at the $\left(dth\right)$ channel. These attention weights $A\left[c\right]$ derived from $S\left(d\right)$ are pivotal for recalibrating the feature responses. As illustrated in Eq. 9, these weights are applied by element-wise multiplying with the batch-normalized output feature maps $Y_{BN}$ , resulting in the recalibrated feature map $Z_{BN}$ . This step is crucial for enhancing the network’s focus on pertinent features within the data.


(9) $$Z_{BN}\;\left[i,j,k,c\right]=A\left[c\right]\times Y_{BN}\;\left[i,j,k,c\right]$$

Subsequent to recalibration, the SAEB’s final output V is generated by applying an activation function ReLU to the recalibrated feature map $Z_{BN}$ , as formulated in Eq. 10. This transformation introduces non-linearity, enabling the extraction of complex patterns from the recalibrated feature map and preparing the model for further processing layers.


(10) $$V\;\left[i,j,k,c\right]=\max\;\left(0,Z_{BN}\;\left[i,j,k,c\right]\;\right)$$

The SAEB final output V is a recalibrated version of the initial input feature map based on channel-wise attention mechanism. This process allows the model to focus on the more relevant features of the task at hand. The model is then used as the final output as the input to the next layer. SAEB recalibrates its output feature maps by using attention weights. This technique allows the model to focus on areas of interest and provide contextually relevant information. In time-sensitive clinical settings or with limited computing resources, this model excels at doing rapid initial screenings.

#### 3.2.2 Dual Adaptive Encoder Block

**Figure 4:**
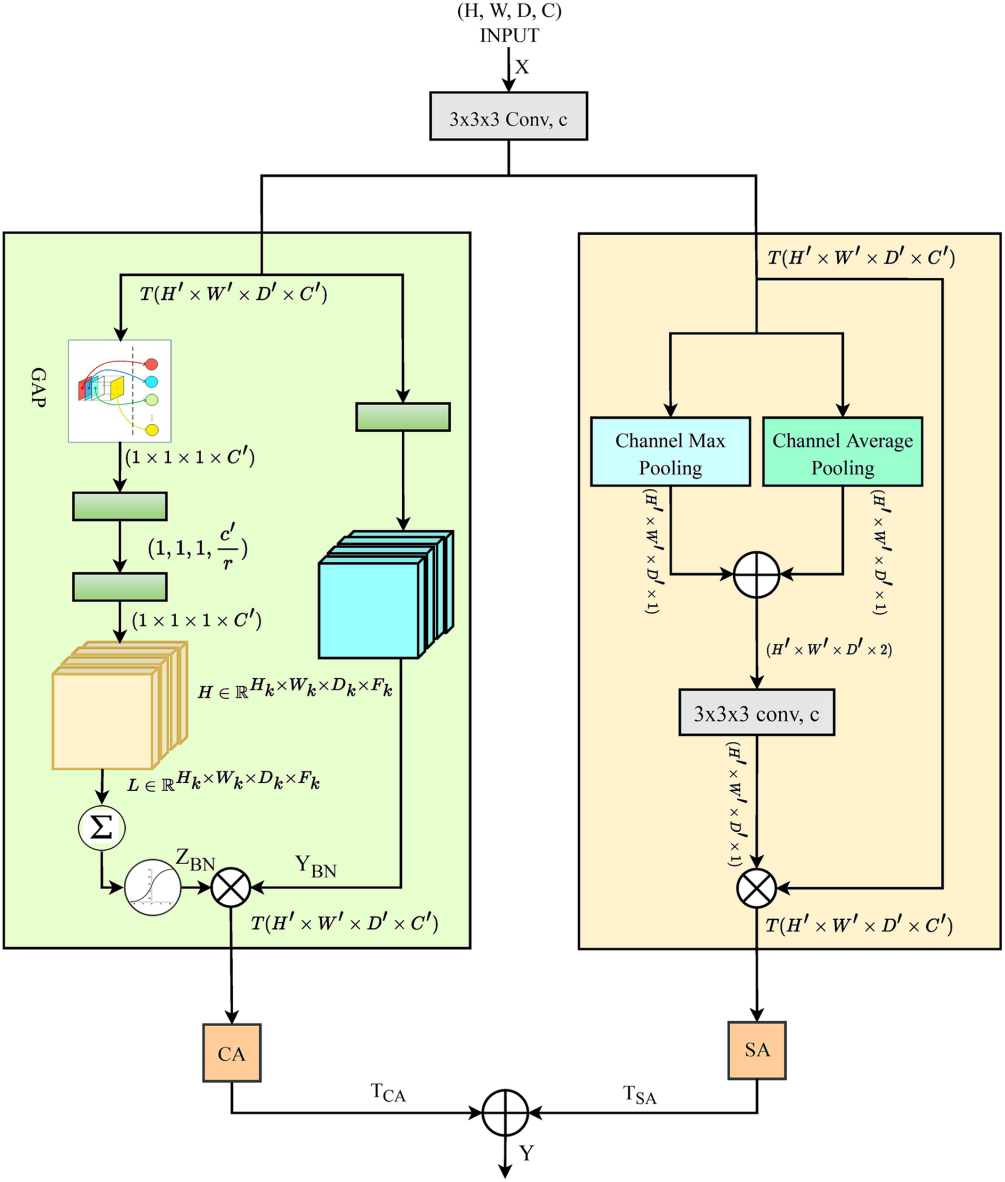
The architecture of the proposed DAEB.

In 3D data processing, Deep learning algorithms present substantial challenges in 3D data processing, such as extracting prominent spatial and channel dimension features. Primarily, CNN models relied heavily on typical convolution operations and activation functions, which frequently fail to highlight the most critical regions of interest within the data. Attention approaches have emerged as practical solutions that focus on more significant features dynamically. Among these attention methods, Hu et al. (2018) introduced Squeeze-and-Excitation (SE) attention, which plays a critical role in recalibrating channel-wise elements of data. This technique is effective but overlooks the spatial dependencies within feature maps. To address this problem, Woo et al. (2018) proposed spatial attention mechanisms, further refined by Li et al. (2020). However, these approaches largely neglect the interaction between channel-wise dependencies. This oversight reveals a compelling opportunity: by integrating both channel-wise and spatial dependencies, model performance could be significantly enhanced. Recognizing this potential, we introduced the DAEB as a proposed solution. The DAEB presents a dual-attention mechanism that significantly extends the suggested network’s capability to highlight fine-grain semantic features. By applying channel-specific and spatial attention mechanisms, the DAEB module offers a comprehensive approach to feature refinement. This dual attention is achieved through the integration of global average-pooled information and subsequent convolutional layer processing, which ensure a more focused and relevant feature map. A visual representation of the DAEB and its operations is shown in Figure 4.

We start by applying a 3D convolution operation, denoted by the function F , to the input tensor X , where $X\in {\mathbb{R}}^{\left(H\times W\times D\times C\right)}$ and H , W , D , and C represent the height, width, depth, and channel dimensions of the tensor, respectively. This operation transforms X into an intermediate tensor T , and the transformation can be denoted as:


(11) $$\begin{array}{l} T\left(i^{\prime },j^{\prime },k^{\prime },c^{\prime }\right)=\\ \;\underset{i_{n}}{\sum }\;\underset{j_{n}}{\sum }\underset{k_{n}}{\sum }\underset{k}{\sum }\;W\left(i,j,k,c,c^{\prime }\right)\times X\left(i^{\prime }+i_{n},j^{\prime }\;+j_{n},k^{\prime }+k_{n},c\right) \end{array}$$

In Eq. 11, the variables $\left(i^{\prime },j^{\prime },k^{\prime },c^{\prime }\right)$ represent coordinates in the new tensor T , and the non-primed ones $\left(i,j,k,c\right)$ represent coordinates in the original tensor X . The variables $\left(i_{n},\;j_{n},k_{n}\right)$ iterate over the kernel dimensions and W represents the kernel weights. This operation extracts localized features from the input tensor X based on the filter weights. Subsequently, we introduce a channel-wise focus through the GAP mechanism, which is applied to the tensor T . This yields the global descriptor $CA\in R^{C^{\prime }}$ , where $C^{\prime }$ representing the channels in the transformed tensor:


(12) $$CA\left(c^{\prime }\right)=\left(\frac{1}{\left(H^{\prime }\times W^{\prime }\times D^{\prime }\right)}\right)\times \underset{i}{\sum }\underset{j}{\sum }\underset{k}{\sum }T\left(i,j,k,c^{\prime }\right)$$

In Eq. 12, ( $H^{\prime }$ , $W^{\prime }$ , $D^{\prime }$ ) represents the height, width, and depth dimensions of T , respectively. The global descriptor CA gives importance to informative channels and suppresses the less relevant ones in tensor T . Then, two-step transformation process is implemented on the global descriptor CA , yielding a new descriptor CA′ :


(13) $$\begin{array}{l} CA\prime \left(i^{\prime },j^{\prime },k^{\prime },c^{\prime \prime }\right)=\\ \;\underset{i_{n}}{\sum }\underset{j_{n}}{\sum }\underset{k_{n}}{\sum }\underset{k}{\sum }\;W^{\prime }\left(i,j,k,c^{\prime },c^{\prime \prime }\right)\times CA\;\left(i^{\prime }+i_{n},j^{\prime }+j_{n},k^{\prime }+k_{n},c^{\prime }\right) \end{array}$$

In Eq. 13, $W^{\prime }$ represents the transformation weights, and the $c^{\prime \prime }$ term denotes the channels in the newly transformed descriptor. This transformation helps to highlight channel-wise dependencies in the global descriptor CA .


(14) $$CA\left(i^{\prime },j^{\prime },k^{\prime },c^{\prime \prime }\right)=\sigma \left(CA\prime \;\left(i^{\prime },j^{\prime },k^{\prime },c^{\prime \prime }\right)\right)$$

The transformation process is further refined by applying a sigmoid activation function $\left(\sigma \right)$ to the descriptor CA′ , which generates the channel-wise attention map CA as detailed in Eq. 14, effectively scaling each channel’s values within the interval [0, 1]. This step is essential for determining the significance of each channel in terms of the spatial features of the input tensor. After obtaining the channel-wise attention map CA , reweight the tensor T through an element-wise multiplication operation, yielding tensor $T_{CA}$


(15) $$T_{CA}\left(i^{\prime },j^{\prime },k^{\prime },c^{\prime }\right)=\;T\;\left(i^{\prime },j^{\prime },k,c^{\prime }\right)\times CA\;\left(i^{\prime },j^{\prime },\;k^{\prime },c^{\prime \prime }\right)$$

Eq. 15 describes the application of the channel-wise attention map CA , where the recalibrated tensor $T_{CA}$ is produced by an element-wise multiplication with the tensor T . This operation enables the model to adaptively emphasize informative features and suppress irrelevant ones in the tensor T . Then, we compute the spatial attention map $SA\in {\mathbb{R}}^{\left(H^{\prime }\times W^{\prime }\times D^{\prime }\times 2\right)}$ as illustrated in Eq. 16, by concatenating the maximum and average pooling maps derived from T .


(16) $$SA=\left[\mathrm{MaxPool}\left(T\right)⊕\mathrm{AvgPool}\left(T\right)\right]$$

This step captures spatial dependencies in the feature maps. The spatial attention map SA is then transformed through a 3D convolution operation denoted by Conv .


(17) $$SA=\mathrm{Conv}\left(SA\right)$$

In Eq. 17, the spatial attention map SA undergoes a 3D convolution transformation, which enhances the model’s capability to capture spatial dependencies within the feature maps. This convolution operation consolidates the various spatial features into a more coherent structure that is crucial for accurate segmentation. Following this convolution, Eq. 18, details how the spatial attention map SA scaled by a sigmoid activation function, assigning a value between 0 and 1 to each position. This scaling effectively ranks the spatial features by their relevance. The resulting map is then utilized to modulate the tensor T , with an element-wise multiplication producing the reweighted tensor $T_{SA}$ .


(18) $$T_{SA}=T⊗SA$$

The process, affiliated with channel-wise reweighting, allows the model to emphasize informative features and suppress irrelevant ones adaptively. Finally, we combine the outputs of the channel-wise and spatial attention mechanisms applied separately to the input tensor. The resultant tensors (not the attention maps) are fused to generate the final output tensor Y :


(19) $$Y=T_{CA}⊕T_{SA}$$

Eq. 19 represents the fusion of the channel-wise and spatial attention mechanisms, resulting in the new output tensor Y . The model leverages informative channels and spatially relevant regions by integrating these outputs, thereby effectively understanding and classifying complex multi-dimensional data. The DAEB’s dual-attention mechanism addresses the need for extracting prominent features across both spatial and channel dimensions, effectively overcoming the limitations of traditional CNN models that may overlook critical regions of interest within the data. By implementing the DAEB, it is anticipated that models can learn more effectively from 3D data, potentially leading to enhanced performance across various tasks and domains. The DAEB consistently outperformed existing models through rigorous experimental analysis, solidifying its standing as an optimized solution for 3D data segmentation.

### 3.3 SegPath

Semantic segmentation has various approaches for enhancing the connectivity between encoders and decoders. In this regard, the skip connection is an outstanding solution that has gained recognition, particularly in architectures such as U-Net. This method enables encoder features to be directly associated with corresponding decoder layers, thereby ensuring the preservation and recovery of spatial details, which is vital for accurate segmentation. Merging the encoder features (low-level features) with decoder features (high-level features) results in a semantic gap.

**Figure 5:**
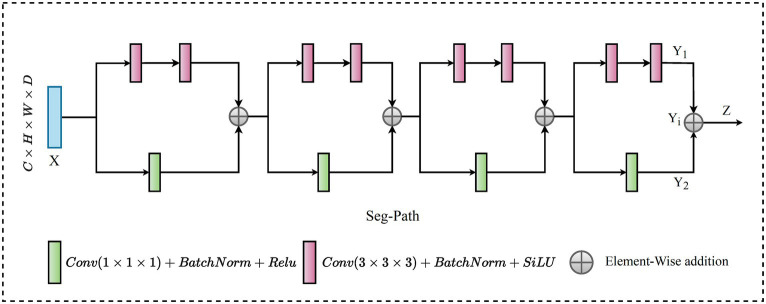
The framework of SegPath.

In recent research on connectivity strategies, the ResPath architecture emerged, integrating residual connections reminiscent of the ResNet strategy within the skip pathways. This fusion improves the model’s ability to learn refined residual feature representations. Moreover, Mubashar et al. (2022) combines dense and skip connections in a significant way. This architecture ensures that all feature maps are densely connected via a skip connection structure. Drawing from these improvements, we present the SegPath module, a sophisticated modification to the skip connection structure, as shown in Figure 5. SegPath enhances segmentation performance through two fundamental processes: adaptive feature accumulation and the integration of multi-scale contextual information. Adaptive feature accumulation works by iteratively accumulating enhanced feature maps through element-wise addition, enabling SegPath to form a comprehensive representation of the input data. This process allows for the adaptive refinement of feature maps, customized to meet the specific requirements of the segmentation task. Concurrently, SegPath employs parallel transformations to capture a wide range of aspects from the input feature map, including detailed textures and broader contextual information. These transformations involve convolving the input feature map with filters of different sizes (1 × 1 × 1 and 3 × 3 × 3), followed by batch normalization and ReLU activation. It incorporates a series of parallel transformations to capture various aspects of the input feature map X . In the first transformation, X undergoes a 1 × 1 × 1 convolution with a filter $F_{1}$ , resulting in a tensor $X_{1}$ . This operation can be formulated as shown in Eq. 20,


(20) $$X_{1}\left(i,j,k,l\right)=\underset{m}{\sum }X\left(i,j,k,m\right)\times F_{1}\left(1,1,1,m\right)$$

In Eq. 20, i , j and, k are spatial locations in the 3D feature maps and 1 denotes the feature channel at each spatial location. The index m is used to iterate over the feature channels in the input feature map X and the convolution filter $F_{1}$ . Simultaneously, X is convolved with a 3×3×3 filter $F_{2}$ , leading to tensor $X_{2}$ , as expressed in Eq. 21.


(21) $$\begin{array}{l} X_{2}=\left(i,j,k,l\right)=\;\underset{m}{\sum }\underset{a=-1^{1}}{\sum }\underset{k=-1^{1}}{\sum }\underset{x=-1^{1}}{\sum }\;X\;\left(i+a,j+b,k+c,m\right)\\ \;\times F_{2}\;\left(a+2,b+2,c+2,m\right) \end{array}$$

where a , b , c used to traverse the 3D convolution filter’s spatial extent during the convolution operation, ranging from −1 to 1 to cover the 3×3×3 spatial extent of the filter $F_{2}$ . After each convolution, batch normalization is applied to normalize the tensor, creating $X_{1}$ normalized and $X_{2}$ normalized tensors. Following the normalization step, the ReLU activation function is applied element-wise to $X_{1}$ and $X_{2}$ normalized, resulting in tensors $Y_{1}$ and $Y_{2}$ , respectively, detailed in Eq. 22 and Eq. 23.


(22) $$Y_{1}\left(i,j,k,l\right)=\max\left(0,X_{1}\mathrm{Norm}\left(i,j,k,1\right)\right)$$

and,


(23) $$Y_{2}\left(i,j,k,l\right)=\max\left(0,X_{2}\mathrm{Norm}\left(i,j,k,1\right)\right)$$

These enhanced feature maps $Y_{1}$ and $Y_{2}$ are accumulated through element-wise addition to create an enhanced representation $Y_{i}$ for each iteration i , as outlined in Eq. 24.


(24) $$Y_{i}\left(i,j,k,l\right)=Y_{1}\left(i,j,k,1\right)+Y_{2}\left(i,j,k,1\right)$$

This step is repeated n times, where $\left[i=1,2,\ldots ,n\right]$ and the outputs are summed together to obtain the final output tensor Z , encapsulated in Eq. 25.


(25) $$Z=\overset{n}{\underset{i=1}{\sum }}Y_{i}$$

The accumulation of adaptive features enriches the information carried by the final output tensor, Z allowing for better capture of complex patterns and variations in the input data. This is particularly crucial in medical image analysis tasks, where detailed and accurate feature extraction is key to successful segmentation. This approach ensures a general understanding of the samples, significantly improving segmentation outcomes by using the strengths of both detailed and contextual information processing within the model. The adaptive feature accumulation of the SegPath block allows for learning more critical features for the specific task, thus enhancing its representative capacity. Furthermore, it provides an additional path for gradient flow through the adaptive features, improving the mitigation of the vanishing gradient problem.

## 4 Materials and experimental setup

### 4.1 Materials

To demonstrate the broad utility and effectiveness of our proposed model, AFMS-Net, we have used three appreciated, publicly accessible datasets, each supporting a distinct medical image segmentation task. The details of these datasets are summarized in Table 1. Specifically, the Brain Tumor Segmentation BRATS2021 dataset (Baid et al., 2021), facilitates brain tumor segmentation. For ischemic stroke lesion identification and tracing of lesions after a stroke, we have employed the Anatomical Tracings of Lesions after Stroke (ATLAS v2.0) 2021 datasets (Liew et al., 2017), and the Ischemic Stroke Lesion Segmentation (ISLES) 2022 datasets (Hernandez Petzsche et al., 2022), respectively. In addition, we used rigid registration and affine transformation techniques to register ISLES datasets according to the standard Montreal Neurological Institute (MNI) space (Chau and McIntosh, 2005).

**Table 1:** Details of the medical segmentation datasets used in our experiments.

Dataset	Images	Voxel size	Input size	Train	Valid	Test
BraTS 2021	1,151	1 × 1 × 1	128 × 128 × 128	874	115	162
ATLAS v2.0	655	1 × 1 × 1	160 × 160 × 160	458	105	092
ISLES 2022	246	2 × 2 × 2	128 × 128 × 128	196	024	026

#### 4.1.1 Brain tumor segmentation datasets

**Figure 6:**
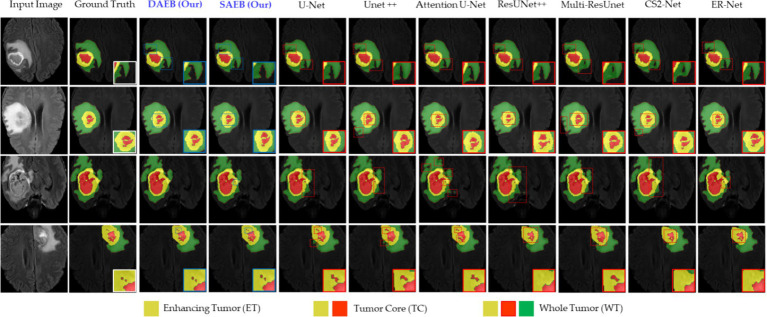
Illustrative examples highlighting diverse challenges in the BraTS Dataset. The set of samples is organized from left to right as follows: the original input image, the associated ground truth, and the segmentation outputs generated by our models (SAEB and DAEB), U-Net, Unet++, Attention U-Net, ResUNet++, Multi-ResUnet, CS2-Net, and ER-Net. Four distinct samples demonstrate specific challenges: boundary delineation, fine-grained analysis, and lesion variability. This comprehensive comparison aims to underscore each segmentation technique’s relative strengths and limitations in addressing these challenges.

The Proposed framework utilized the BraTS-2021 benchmark dataset, which includes a training set comprising 1,251 patients with both High-Grade Gliomas (HGG) and Low-Grade Gliomas (LGG). Each patient dataset consists of four MRI sequences: T1-weighted (T1), contrast-enhanced T1-weighted (T1ce), T2-weighted (T2), and fluid-attenuated inversion recovery (FLAIR). These sequences offer a detailed and multidimensional view of the tumor, aiding in more precise segmentation. Images in the dataset were collected following various clinical guidelines, using MRI machines of differing specifications and magnetic intensities, contributing to its heterogeneity. The image preprocessing steps were critical in ensuring data consistency across all datasets. It included co-registration of each patient’s MRI modalities, skull stripping, and voxel resampling to a 1 mm^3^ isotropic resolution, resulting in a uniform MRI volume size of 155 × 240 × 240. The ground truth segmentation for each MRI volume was categorized into four segments: background, Necrotic and Non-enhancing Tumor (NCR), Peritumoral Edema (ED), and Enhancing Tumor (ET). However, for evaluation, the three nested sub-regions, namely enhancing tumor (ET), tumor core (TC—i.e., the union of ED and NCR/NET), and whole tumor (WT), are used (see the sample ground truths in Figure 6). In order to enhance computational efficiency and concentrate the suggested model’s attention on the most pertinent areas, resized the original volume to dimensions of 128 × 128 × 128.

Moreover, we fused the FLAIR, T1ce, and T2 modalities into a single multi-channel image, which provided proposed framework with the most comprehensive information about each tumor’s characteristics. In data preprocessing step, we implemented a filtering mechanism to disregard less informative samples. Specifically, any volume where less than 1% of labels were non-zero (indicative of tumor presence) was deemed “useless” and discarded. This helped reduce noise in the training data, thereby enhancing the learning efficiency of our model. For a comprehensive model evaluation, we systematically divided the data by allocating 80% for model training, allowing the model to learn from diverse information. The remaining 20% was equally divided into validation and test sets. The validation set helped fine-tune our model’s hyper-parameters. In contrast, the test set assessed our model’s performance on unseen data, providing a more reliable evaluation of its effectiveness.

#### 4.1.2 ATLAS v2.0 dataset

The ATLAS v2.0 dataset, a meticulously composed repository of MRI scans and lesion segmentation masks, has been methodically organized into three subsets: training, testing, and a holdout set. The training subset comprises 655 T1-weighted MRI scans from multiple cohorts, each linked with its corresponding lesion segmentation mask. The test subset includes 300 T1-weighted MRI scans drawn from the same cohorts, with their respective lesion segmentation masks intentionally hidden. The holdout test set encapsulates 316 entirely obscured T1-weighted MRI scans and lesion segmentation masks, each originating from an independent set. This dataset was utilized strategically through a comprehensive preprocessing pipeline in the experimental process. The initial step involved performing a central cropping operation on the image data to a size of 160 × 160 × 160 voxels. Focusing on the region of interest reduced superfluous peripheral information, thereby enhancing computational efficiency. Standardizing voxel size across the dataset involved resampling the cropped image data, contributing to consistent and reliable outcomes in subsequent machine-learning tasks. The image data was normalized to diminish the impact of intensity variations across different MRI scans. Gaussian smoothing was implemented to mitigate the influence of noise on the MRI scans. This technique not only reduced noise but also augmented the visibility of the lesions, thereby improving detection accuracy. Simultaneously, lesion segmentation masks were resampled to match the size of the corresponding image and converted into a one-hot encoded format, facilitating their integration into subsequent machine-learning tasks. The 655 T1-weighted MRI scans were then divided into training, validation, and testing sub-sets, comprising approximately 70, 16, and 14% samples, respectively. This stratified splitting strategy balanced the representation of different lesion sizes across all subsets, circumventing potential bias in the model training phase. This rigorous approach guarantees the validity and robustness of the experimental procedures.

#### 4.1.3 ISLES 2022 dataset

The ISLES dataset is designed to evaluate automated acute and subacute stroke lesion segmentation methods in 3D multi-modal MRI data. For our experiments, we used a series of preprocessing steps. The dataset consists of DWI, ADC, and FLAIR images. The FLAIR image was registered to the standard Montreal Neurological Institute (MNI) space (Chau and McIntosh, 2005) using an affine transformation, creating a transformation matrix. This transformation matrix was then used to register the DWI and ADC images first to the original FLAIR images and then to the standard MNI space. In other words, the FLAIR image was registered to the DWI space utilizing rigid registration and affine transformation techniques. After registration, the ADC, DWI, and FLAIR data were consolidated into a multi-channel image. Each image was cropped to a size of 128 × 128 × 128, improving computational efficiency by removing non-essential regions. The dataset encompasses a total of 246 samples. To ensure an unbiased evaluation of our developed model, we randomized the data and divided it into training, validation, and testing sets, adhering to an 80-10-10 split.

### 4.2 Experimental setup

The proposed approach was implemented and trained using the TensorFlow and Keras frameworks, and all experiments conducted on NVIDIA RTX A5000 GPUs. This setup offered the computational power necessary for handling the intensive demands of training deep learning models on complex medical image datasets. The choice of hardware reflects a balance between computational efficiency and the capability to process large volumes of data, characteristic of medical imaging tasks.

#### 4.2.1 Model optimization and hyperparameter selection

Our experimental strategy employment the Adam optimizer, chosen for its effectiveness in handling sparse gradients and adaptively adjusting learning rates, which is crucial for deep learning applications in medical imaging. We set the learning rate to a modest 0.0001, a decision informed by preliminary trials that indicated it as optimal for balancing training speed with convergence stability. Similarly, a weight decay of 0.0005 was applied as a regularization measure to mitigate the risk of overfitting—a common challenge in deep learning models. This weight decay introduces a minor penalty to the loss function, proportional to the L2 norm of the model weights, encouraging the model to learn more generalizable features.

#### 4.2.2 Computational resources and model complexity

**Figure 7:**
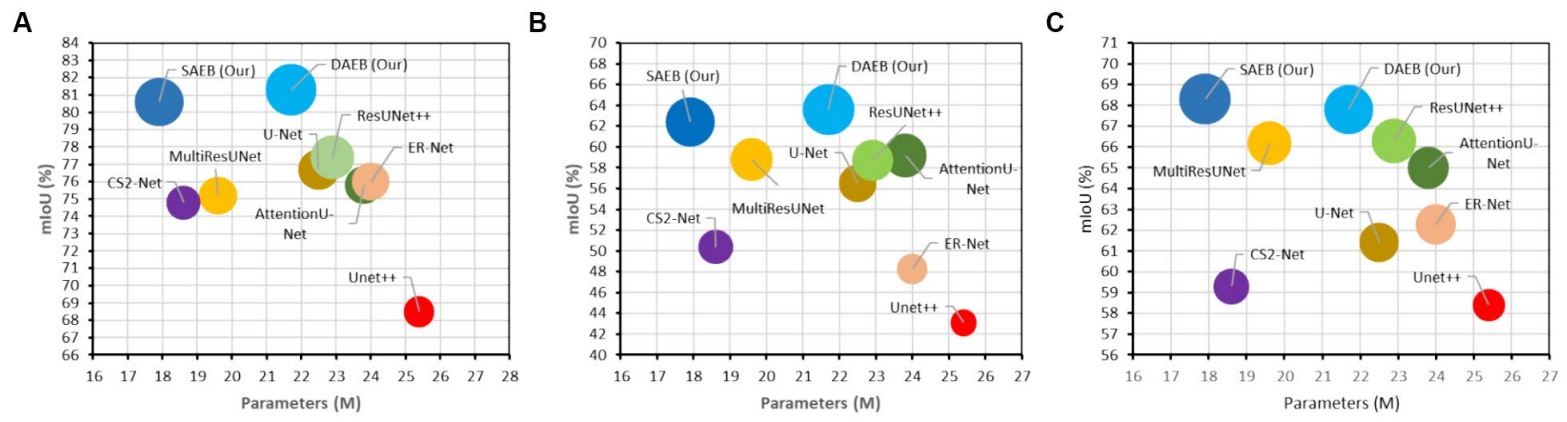
Computational performance trade-offs illustrated by mIoU versus the number of parameters of various models across multiple datasets. **(A)** BraTS 2021, **(B)** ATLAS, and **(C)** ISLES 2022.

Training the AFMS-Net required significant computational resources. Specifically, the training process was executed over approximately 8–16 h on NVIDIA RTX A5000 GPUs, utilizing around 16GB of GPU memory per model instance. These figures highlight the computational demands of training AFMS-Net, emphasizing the need for powerful hardware to achieve optimal performance. To provide a comparative insight into AFMS-Net’s model complexity versus traditional segmentation networks, we reference Figure 7, which illustrates the computational performance trade-offs by comparing mIoU with the number of parameters. This comparison reveals that AFMS-Net achieves a commendable balance between model complexity and segmentation performance. Unlike traditional segmentation networks such as U-Net and its variants, AFMS-Net demonstrates enhanced computational efficiency, achieving competitive or superior performance metrics with a reduced number of parameters. This efficiency is pivotal for deploying advanced segmentation models in real-world medical imaging scenarios, where computational resources might be limited.

#### 4.2.3 Custom loss function

A distinctive feature of our experimental setup is the incorporation of a custom loss function that combines dice loss and categorical focal loss. This approach was designed to address the challenges of class imbalance and ensure accurate segmentation across varying medical image characteristics. The Dice loss, formulated in Eq. 26, is particularly effective in promoting overlap between the predicted segmentation maps and the ground truth, thereby enhancing the model’s precision in delineating lesion boundaries.


(26) $$L_{\mathrm{Dice}}\left(G,P\right)=1-\frac{2\left(\sum_{c}\sum_{i}\;w_{c}\;G_{ci}P_{ci}\right)+\in }{\left(\sum_{c}\sum_{i}\;w_{c}\;G_{ci}+\sum_{c}\sum_{i}w_{c}P_{ci}\right)\;+\in }$$

In Eq. 26, G and P are the ground truth and predicted probability map, c denotes each class, i stands for individual voxels, $w_{c}$ refers to the weight of each class, and ∈ is a small constant used to prevent division by zero.

Further refining the model’s predictive accuracy, the categorical focal loss—described in Eq. 27, adjusts the model’s focus towards difficult-to-classify examples, thereby improving overall classification accuracy.


(27) $$L_{\mathrm{focal}}\left(G,P\right)=-\underset{c}{\sum }\underset{i}{\sum }\;G_{ci}\log\left(P_{ci}\right){\left(1-P_{ci}\right)}^{\gamma }$$

where $P_{ci}$ represents the model’s estimated probability for the true class, γ is a tuning parameter (typically set at 1.0), and the sum is calculated over all classes. Each class was assigned an equal weight for dice loss calculation. Ultimately, the total loss utilized for training the model is computed as the sum of the Dice loss and the categorical focal loss, as shown in Eq. 28. This combined loss function leverages the strengths of both components to provide a balanced optimization criterion.


(28) $$L_{\mathrm{total}}\left(G,P\right)=L_{\mathrm{Dice}}\left(G,P\right)+L_{\mathrm{focal}}\left(G,P\right)$$

### 4.3 Evaluation metrics

This section outlines the key metrics used to assess the model’s effectiveness comprehensively. The proposed brain lesion segmentation model is rigorously evaluated using a comprehensive set of metrics, all at a threshold of 0.5, to provide a thorough understanding of its performance. Accuracy is calculated as the proportion of true predictions, both correct lesion identifications (true positives) and correct non-lesion identifications (true negatives), over the total number of cases, as specified in Eq. 29.


(29) $$\mathrm{Accuracy}=\frac{TP+TN}{TP+TN+FP+FN}$$

where TP are true positives, TP are true negatives, FP are false positives, and FN are false negatives. Precision, defined as the ratio of true positives to the sum of true positives and false positives, reflects the model’s accuracy in predicting lesion instancesinstances, outlined in Eq. 30.


(30) $$\mathrm{Precision}=\frac{TP}{TP+FP}$$

Meanwhile, Recall measures the model’s ability to identify all actual lesion cases, calculated as the ratio of true positives to the sum of true positives and false negatives, as depicted in Eq. 31.


(31) $$\mathrm{Recall}=\frac{TP}{TP+FN}$$

The Dice Score (DSC) expressed in Eq. 32, is used to measure the similarity between the predicted segmentation and the ground truth. It is particularly useful for evaluating models where the class distribution is imbalanced. The DSC is calculated as:


(32) $$DSC=\frac{2TP}{2TP+FP+FN}$$

Intersection over Union (IoU), presented in Eq. 33, also known as the Jaccard index, measures the overlap between the predicted segmentation and the ground truth. It is defined as:


(33) $$IoU=\frac{TP}{TP+FP+FN}$$

The average Hausdorff distance (AHD), uniquely considering voxel location and defined as:


(34) $$AHD=\frac{1}{2}\left(\frac{1}{P}\underset{p\in P\;}{\sum }\min_{l\in L}\;d\left(p,l\right)+\frac{1}{L}\underset{l\in L\;}{\sum }\min_{p\in P}\;d\left(p,l\right)\right)$$

In Eq. 34, P represents the point set of segmentation results, and L denotes the point set of labels, enabling reflection on the edge error of segmentation results. These metrics provide a balanced and comprehensive assessment of the efficacy of the suggested framework in brain lesion segmentation.

## 5 Experimental results

### 5.1 Comparative segmentation performance on diverse datasets

In this section, we present a thorough comparison between seven different state-of-the-art 3D MIS techniques and our suggested approach for brain lesion segmentation. We compare our approach with U-Net, Unet++ (Zhou et al., 2019), AttentionU-Net, ResUNet++ (Jha et al., 2019), Multi-ResUNet (Ibtehaz and Rahman, 2020), CS2-Net (Mou et al., 2021) and ER-Net (Xia et al., 2022). We follow a uniform protocol across all methodologies to ensure a fair and comprehensive comparison. Every baseline model follows the default settings specified by their respective original authors. The structure of each model is based either on the associated codes available on GitHub or descriptions provided by the original authors. We also maintain consistency in preprocessing and post-processing steps across all models. This standardization eliminates potential bias, ensuring the comparative results accurately reflect the performance of each method.

#### 5.1.1 Qualitative and quantitative results on BraTS 2021 dataset

In this section, we evaluate the performance of SAEB and DAEB models on the BraTS 2021 dataset. The qualitative results, illustrated in Figure 6, offer visual insights into the performance of various segmentation methods—the first-row centers on the model’s proficiency in edge detection within tumors. Certainly, most approaches perform similarly well in distinguishing important regions of enhancing tumor (ET), tumor core (TC), and whole tumor (WT). However, differences become noticeable when defining the edges of the tumor. Selected comparison methods, such as U-Net, Unet++, Attention U-Net, ResUnet++, Multi-ResUnet, CS2-Net, and ER-Net, effectively detect larger tumor structures but falter when identifying precise edges. This results in noticeable under-segmentation or over-segmentation. In comparison, The SAEB and DAEB models precisely outline the tumor edges. The blue-red, dotted rectangles and their magnified views highlight the differences. The second row demonstrates the proficiency of SAEB and DAEB in recognizing intricate tumor sub-structures. In contrast, notable methods like U-Net and its variants misrepresent subtle elements such as necrosis or non-enhancing tumor cores. The third row of Figure 6 illustrates the ability of the SAEB and DAEB to emphasize the uniformity of regions within the tumor while simultaneously identifying subtle variations in texture. In the fourth row, we address the fine-grained analysis problem. Interestingly, all the baseline approaches failed to identify these tiny features. However, the suggested framework can identify minute structures and lesions. The visualizations demonstrate the proposed models’ adaptability and precision, highlighting their ability to tackle the intricate challenges presented by the BraTS dataset. For the detailed quantitative analysis, this work is divided into two main sections: overall segmentation performance, presented in Table 2, and segmentation by tumor regions, illustrated in Table 3; this comprehensive analysis evaluates the proposed framework’s effectiveness. The evaluation metrics in Table 2 reinforce the superior performance of our proposed models. Our AFMS-DAEB registers impressive results with an accuracy of 99.01%, precision of 90.80%, recall of 89.06%, DSC of 90.20%, mIoU of 82.23%, and an AHD value of 6.079, respectively. These metrics indicate an approximate 1% enhancement in DSC and mIoU over the AFMS-SAEB model.

**Table 2:** Performance metrics of various methods evaluated on 1,251 cases from the Brats 2021 dataset.

Method	Accuracy	Precision	Recall	DSC	mIoU	AHD
U-Net	0.988 ± 0.003	0.870 ± 0.101	0.865 ± 0.178	0.867 ± 0.123	0.767 ± 0.165	7.010
Unet++	0.927 ± 0.031	0.747 ± 0.187	0.891 ± 0.165	0.812 ± 0.157	0.685 ± 0.176	12.146
AttentionU-net	0.986 ± 0.022	0.899 ± 0.153	0.883 ± 0.127	0.860 ± 0.179	0.758 ± 0.181	8.725
ResUNet++	0.988 ± 0.020	0.882 ± 0.148	0.889 ± 0.186	0.869 ± 0.158	0.774 ± 0.161	6.916
MultiResUNet	0.985 ± 0.025	0.848 ± 0.183	0.868 ± 0.121	0.856 ± 0.153	0.752 ± 0.158	9.125
CS2-Net	0.985 ± 0.033	0.853 ± 0.141	0.858 ± 0.116	0.855 ± 0.172	0.748 ± 0.187	10.165
ER-Net	0.987 ± 0.025	0.860 ± 0.161	0.866 ± 0.194	0.861 ± 0.145	0.761 ± 0.209	8.126
SAEB (Our)	0.989 ± 0.026	0.913 ± 0.118	0.885 ± 0.171	0.894 ± 0.123	0.806 ± 0.117	6.266
DAEB (Our)	0.990 ± 0.031	0.908 ± 0.189	0.896 ± 0.132	0.902 ± 0.151	0.813 ± 0.195	6.079

**Table 3:** Comparative performance metrics for whole tumor (WT), tumor core (TC), and enhancing tumor (ET) in 1,251 cases from the Brats 2021 dataset.

Model	Whole tumor					Tumor core					Enhancing tumor				
	ACC	PRE	REC	DSC	IoU	ACC	PRE	REC	DSC	IoU	ACC	PRE	REC	DSC	IoU
U-Net	0.98	0.89	0.85	0.87	0.77	0.98	0.81	0.84	0.85	0.74	0.98	0.86	0.71	0.79	0.66
Unet++	0.92	0.84	0.81	0.82	0.70	0.92	0.29	0.55	0.45	0.29	0.92	0.83	0.69	0.75	0.60
AttentionU-Net	0.98	0.74	0.87	0.80	0.67	0.98	0.83	0.85	0.84	0.73	0.98	0.86	0.79	0.82	0.70
ResUNet++	0.98	0.85	0.87	0.86	0.76	0.98	0.79	0.83	0.84	0.73	0.98	0.90	0.74	0.81	0.68
MultiResUnet	0.98	0.81	0.84	0.83	0.71	0.98	0.73	0.87	0.81	0.68	0.98	0.82	0.79	0.81	0.68
CS2-Net	0.98	0.89	0.84	0.86	0.76	0.98	0.80	0.84	0.84	0.73	0.98	0.83	0.71	0.78	0.65
ER-Net	0.98	0.89	0.85	0.87	0.77	0.98	0.81	0.84	0.85	0.74	0.98	0.85	0.74	0.79	0.66
SAEB (Our)	0.98	0.91	0.85	0.88	0.79	0.98	0.86	0.85	0.85	0.75	0.98	0.87	0.78	0.83	0.71
DAEB (Our)	0.99	0.84	0.91	0.87	0.78	0.99	0.88	0.86	0.87	0.77	0.99	0.87	0.83	0.85	0.74

When benchmarked against state-of-the-art models, our models exhibit a considerable edge. While U-Net, with its 86.7% DSC and 76.7% mIoU, is commendable, it’s surpassed by AFMS-DAEB, particularly in DSC and mIoU. Unet++ shows room for improvement, especially with its 81.2% DSC. Attention U-Net and ResUNet++ deliver DSC values around 85%, yet are outperformed by our models. Similarly, despite their respective merits, Multi-ResUNet, C2Net, and ErNet fall short compared to AFMS-DAEB’s segmentation efficacy. In principle, AFMS-DAEB not only refines the capabilities of AFMS-SAEB but also delineates itself as a potent tool among established segmentation techniques, showcasing its aptitude for nuanced medical image segmentation. Further examining the segmentation performance across models, we assess three critical tumor categories: WT, TC, and ET, as detailed in Table 3. In the WT segmentation, our AFMS-SAEB model emerges as a front-runner, boasting an accuracy of 98.9%, a precision of 91.3%, a DSC of 88.4%, and mIoU of 79.2%. These metrics showcase the superiority of some models over others. For instance, U-Net achieved a DSC of 87.4% and a mIoU of 77.7%.

On the other hand, Unet++ is behind with a DSC of 45.4%, while AttentionU-Net and ResUNet++ have better results, with mIoU scores of 73.2 and 73.5%. The mIoU score of SAEB is 79.2%, which matches closely with the ground truths. For TC, DAEB has an excellent performance. Its accuracy is 99%, precision is 88.6% and DSC score reaches up to 87.5% and mIoU value of around 77%. For the ET, AFMS-DAEB showcases a commendable DSC of 85.1% and a mIoU score of 74%. Compared to other base models, ER-Net and MultiResUNet demonstrate promising results. In conclusion, based on evaluation metrics, SAEB and DAEB show promising tumor segmentation capabilities. The combination of insights from both tables provides a comprehensive evaluation of each model’s segmentation performance and specialization inside various tumor locations.

#### 5.1.2 Qualitative and quantitative results on ATLAS R2.0 dataset

**Figure 8:**
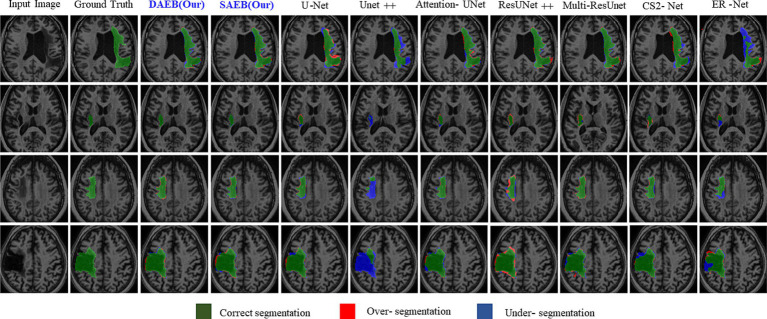
Visual comparison of segmentation challenges in four representative samples from the ATLAS R2.0 dataset. Arranged from left to right are: The original input image, the ground truth, followed by outputs from our models (DAEB, SAEB), U-Net, Unet++, Attention U-Net, ResUNet++, Multi-ResUNet, CS2-Net, and ER-Net. The samples are chosen to highlight distinct challenges inherent to the ATLAS dataset.

Precise lesion segmentation can significantly aid stroke diagnosis and treatment. Our proposed method demonstrates this precision across four diverse stroke cases, which are visually presented in Figure 8. These cases vary in lesion location, shape, and size within the brain, highlighting the adaptability of our approach. In the first row, the lesion is located in the anterior limb and genu of the internal capsule. AFMS-DAEB and AFMS-SAEB, predict almost the entire lesion completely, achieving a remarkable advantage over the benchmark models. While U-Net, Attention U-Net, ResUNet++, and Multi-ResUNet manage to identify most of the lesions, but they tend to over-segment the affected area.

On the other hand, Unet++, C2Net, and ER-Net only delineate a small fraction of the lesion. The second row examine a lesion in the internal capsule’s posterior limb. Here, Unet++ and ER-Net struggle to mark the lesion accurately. U-Net and AttentionU-Net identify only portions of it. Although closer to the mark, ResUNet++, Multi-ResUNet, and CS2-Net present evident over-segmentations. However, the proposed framework captures this lesion clearly, highlighting its adeptness at processing boundary information. In the third row, the lesion, with its regular shape and precise location, presents a more straightforward segmentation target. Both AFMS-Net variants demonstrate superior performance in delineating the lesion accurately.

Among the benchmark models, AttentionU-Net stands out as the most effective for this particular case. Conversely, ResUNet++ and Multi-ResUNet exhibit over-segmentation issues, while the other models tend to under-segment the designated region. The lesion in the fourth row is large and irregular and located near the junction of the central and superior temporal sulcus. Only AFMS-Net adeptly captures previously overlooked regions of all models, ensuring a thorough and accurate segmentation. Meanwhile, the benchmark methods vary, with some showing marked over-segmentation or under-segmentation tendencies. Across all scenarios, Unet++ and ER-Net consistently lean towards conservative segmentations, resulting in substantial under-segmentation. Conversely, ResUNet++ and U-Net tend to produce aggressive segmentation, often mistakenly classifying cerebrospinal fluid in the lateral ventricles as target lesions. While ResUNet++ and Multi-ResUNet demonstrate commendable consistency regarding region similarity and boundary delineation, they do not surpass the benchmark models in all aspects. However, our proposed AFMS-Net excels in identifying areas that benchmark methods either under-segmented or over-segmented, ensuring improved region alignment and enhanced boundary precision. While visual analysis provides insights into segmentation performance, a comprehensive quantitative assessment is essential for conclusive determinations. Accordingly, we subjected our proposed AFMS-Net and other prominent methods to rigorous evaluation metrics, with the detailed outcomes reported in Table 4. In a comparative assessment against prevailing methods, the proposed AFMS-DAEB distinctively achieves an impressive DSC of 78.20% and a mIoU of 63.60%. When benchmarked in mIoU scores, AFMS-DAEB consistently outperforms-surpassing U-Net by 8%, Unet++ by 19%, Attention U-Net by 4.4%, etc. This noticeable edge emphasizes our model’s finesse in lesion segmentation and its proficiency in differentiating lesions from the intricate background noise typically found in medical imaging. In evaluating Precision and Recall, apparent differences emerge among the methods. Unet++ performs notably well in precision, with a score of 86.6%, reflecting its accuracy in detecting true positives.

**Table 4:** Performance metrics of various segmentation methods evaluated on 655 cases from the ATLAS dataset.

Method	Accuracy	Precision	Recall	DSC	mIoU	AHD
U-Net	0.996 ± 0.014	0.727 ± 0.134	0.718 ± 0.154	0.721 ± 0.153	0.565 ± 0.171	11.850
Unet++	0.996 ± 0.025	0.866 ± 0.032	0.460 ± 0.018	0.602 ± 0.014	0.431 ± 0.017	13.798
AttentionU-Net	0.997 ± 0.026	0.782 ± 0.156	0.709 ± 0.123	0.742 ± 0.021	0.592 ± 0.165	11.407
ResUNet++	0.995 ± 0.027	0.769 ± 0.143	0.711 ± 0.154	0.738 ± 0.176	0.587 ± 0.169	12.232
MultiResUNet	0.993 ± 0.027	0.770 ± 0.176	0.713 ± 0.121	0.740 ± 0.153	0.588 ± 0.146	11.621
CS2-Net	0.997 ± 0.028	0.650 ± 0.137	0.692 ± 0.123	0.670 ± 0.175	0.504 ± 0.189	12.950
ER-Net	0.997 ± 0.025	0.716 ± 0.162	0.597 ± 0.175	0.653 ± 0.137	0.483 ± 0.212	13.396
SAEB (Our)	0.997 ± 0.028	0.820 ± 0.117	0.732 ± 0.165	0.772 ± 0.135	0.624 ± 0.102	10.642
DAEB (Our)	0.997 ± 0.034	0.839 ± 0.145	0.736 ± 0.131	0.782 ± 0.136	0.636 ± 0.175	10.416

On the other hand, our AFMS-DAEB leads in the recall, scoring 73.60%, highlighting its ability to detect most lesions effectively. Additionally, the DSC metric, essential for assessing the spatial overlap accuracy between the predicted segmentation and the ground truth, highlights the superior performance of AFMS-DAEB. Specifically, it leads by a 4–5% margin compared to the top benchmark model. Conclusively, these quantitative analyses demonstrate the excellent performance of our proposed network and highlight AFMS-DAEB’s adeptness in complex tasks, notably boundaries and edge detection, a consistent challenge in medical image segmentation.

#### 5.1.3 Qualitative and quantitative results on ISLES 2022 dataset

**Figure 9:**
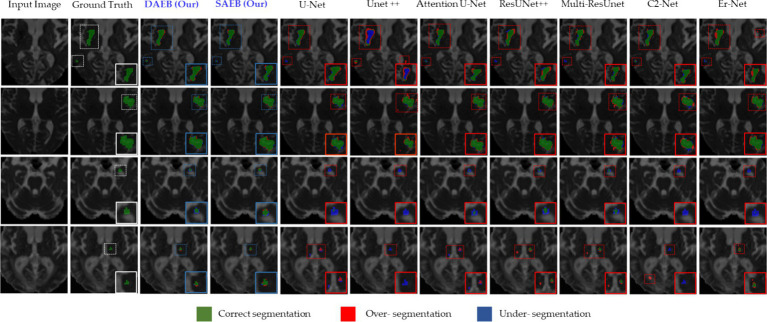
Visual examination of segmentation challenges in four selected samples from the ISLES 2022 Dataset. The arrangement from left to right comprises the original input image, the ground truth, and the segmentation outputs from our models (SAEB, DAEB), U-Net, U-Net++, Attention U-Net, ResUNet++, Multi-ResUnet, CS2-Net, and ER-Net. The samples are specifically chosen to clarify unique challenges such as multiple lesions, small lesions, and varying lesion sizes (median and large).

Similarly to section 5.2, we used the ISLES’22 dataset to evaluate our proposed variants further. This rigorous assessment emphasizes our model’s efficacy (presented in Figure 9). This figure comprises four distinct rows, each corresponding to a specific stroke patient case. These cases encompass a range of complexities, from large infarct lesions to multiple embolic and cortical infarcts, which vary remarkably in location, size, and shape. In the first row, an apparent large lesion is accompanied by a smaller one. A group of benchmark models, specifically U-Net, Unet++, AttentionUnet, ResUnet++, Multi-ResUnet, and ER-Net, failed to accurately segment the minor lesion. However, ER-net and ResUnet++ tended to over-segment, whereas Unet++ could not segment both lesions effectively. The delineated regions of interest are highlighted using a dotted rectangular line, and a zoomed view is provided for enhanced clarity. Ground truths are distinctly represented in white, our proposed models in blue, and benchmark models in red. The second row demonstrates that all segmentation methods identified the lesion’s location. However, some inconsistencies were noted among the benchmark models. U-Net, AttentionUnet, and ResUnet showed tendencies of over-segmentation.

On the contrary, Unet++ and Er-Net leaned towards under-segmentation. In this context, our AFMS-Net demonstrated superior accuracy in delineating the lesion’s shape, achieving remarkable regional overlap. The third and fourth rows present additional challenges, especially concerning smaller lesions. The benchmark models— U-Net, UNet++, AttentionUnet, ResUnet++, Multi-ResUnet, and ER-Net—all struggled with accurately segmenting the minor lesion. In stark contrast, our AFMS-Net showcased its competency by confidently segmenting all lesions, highlighting its distinct advantage in handling diverse lesion types. Quantitative analysis offers an objective perspective on the efficacy of segmentation models. Our evaluation of the ISLES’22 dataset, presented in Table 5, outlines the performance of AFMS-SAEB and AFMS-DAEB compared to other prominent models.

**Table 5:** A comprehensive evaluation of segmentation performance metrics for various methods across 246 cases in the ISLES 2022 dataset.

Method	Accuracy	Precision	Recall	DSC	mIoU	AHD
U-Net	0.994 ± 0.021	0.828 ± 0.130	0.704 ± 0.132	0.761 ± 0.123	0.614 ± 0.193	11.514
Unet++	0.994 ± 0.023	0.806 ± 0.021	0.680 ± 0.014	0.724 ± 0.013	0.584 ± 0.160	15.130
AttentionU-Net	0.995 ± 0.015	0.798 ± 0.130	0.768 ± 0.132	0.778 ± 0.021	0.650 ± 0.224	11.961
ResUNet++	0.995 ± 0.032	0.814 ± 0.132	0.771 ± 0.128	0.789 ± 0.152	0.663 ± 0.213	11.386
MultiResUNet	0.995 ± 0.026	0.843 ± 0.124	0.749 ± 0.121	0.787 ± 0.101	0.662 ± 0.195	12.534
CS2-Net	0.995 ± 0.022	0.775 ± 0.132	0.722 ± 0.123	0.733 ± 0.121	0.593 ± 0.190	13.312
ER-Net	0.995 ± 0.023	0.776 ± 0.136	0.755 ± 0.143	0.760 ± 0.139	0.623 ± 0.240	12.403
SAEB (Our)	0.995 ± 0.025	0.839 ± 0.132	0.780 ± 0.148	0.818 ± 0.136	0.680 ± 0.202	9.855
DAEB (Our)	0.995 ± 0.029	0.860 ± 0.139	0.761 ± 0.138	0.802 ± 0.129	0.673 ± 0.158	10.041

Most models demonstrate an impressive accuracy of around 99.5%, indicating a generally consistent segmentation accuracy across the board. In terms of precision, AFMS-DAEB achieves an outstanding 86.0%, outstripping all other models. Close behind is the Multi-ResUNet, with 84.3%. U-Net and Unet++ demonstrate 82.8 and 80.6% precision scores, respectively. When evaluating recall, the proposed AFMS-SAEB leads with a score of 78.0%. ResUNet++ and AttentionU-Net follow closely with 77.1 and 76.8% recalls, respectively. AFMS-DAEB further asserts its robustness with a recall of 76.1%. The DSC offers a holistic perspective on the overlap between the segmented output and the ground truth. AFMS-SAEB scores 81.8% in DSC, ResUNet++ has a DSC of 78.9%, and AFMS-DAEB reaches up to 80.2%. Regarding mIoU, AFMS-SAEB, which scores 68.0%, AFMS-DAEB closely follows this at 67.3%, and the competing models ResUNet++ and Multi-ResUNet are in the 66% range. In conclusion, each model has its strengths in specific domains. The proposed framework demonstrate an adept balance across all key metrics. This broad examination highlights the proficiency and capabilities of the proposed approach in medical image processing.

#### 5.1.4 Generalizability across different imaging modalities and datasets

Our study mainly focuses on MRI datasets, which are crucial for brain lesion segmentation due to their high resolution and contrast between different brain tissues. We acknowledge the importance of assessing our model’s generalizability across different imaging modalities to ensure its applicability in diverse clinical settings. However, our current investigation is confined to MRI data, considering its relevance and specificity to brain lesion analysis. The datasets utilized in our study encompass a range of MRI images with varying voxel sizes, which are as follows: BraTS 2021 and ATLAS v2.0 datasets have a voxel size of 1 × 1 × 1 mm, providing high-resolution images for precise segmentation. Conversely, the ISLES 2022 dataset has a larger voxel size of 2 × 2 × 2 mm, demonstrating our model’s adaptability to images with lower resolution and potentially different characteristics. By evaluating AFMS-Net across these datasets, we aim to demonstrate its robustness not only to different lesion types but also to variations in image resolution, which is a step toward generalizability. However, we recognize that further studies are necessary to evaluate the model’s performance across other imaging modalities, such as computed tomography (CT) scans or positron emission tomography (PET) images. Future work will involve extending our framework to include these modalities, thereby enhancing its diagnostic versatility and clinical utility.

### 5.2 Ablation studies

In this study, we introduce two encoder modules, SAEB and DAEB, in addition to a SegPath. We propose two different encoders to balance performance efficiency and computational cost. While SAEB offers competitive performance with fewer parameters, DAEB, although computationally more demanding, delivers slightly superior results. To evaluate the effectiveness of these components, we performed ablation studies using one brain tumor dataset and two-stroke datasets, specifically the BraTS 2021, ATLAS R2.0, and ISLES 2022 datasets. Initially, we evaluated the performance impact of substituting the original encoder in the 3D U-Net with our proposed SAEB encoder, resulting in the modified model termed AFMS-SAEB. This adaptation led to incremental gains in DSC and IoU by 0.36 and 0.09% for the BraTS 2021, 0.66 and 0.75% for the ATLAS, and 0.66 and 0.75% for the ISLES 2022. These results can be referenced in Table 6. Motivated by these initial findings, we explored the DAEB encoder as an alternative, creating the AFMS-DAEB model. The DAEB encoder exhibited superior performance, boosting DSC and IoU by 0.96 and 1% on the BraTS 2021, 1.3 and 1.5% on the ATLAS, and 1.5 and 2.7% on the ISLES 2022 dataset. These enhancements are also detailed in Table 6. Aside from qualitative improvements in segmentation, we also examined the computational performance of our proposed models. A comparative analysis between mIoU and the number of parameters for AFMS-SAEB and AFMS-DAEB and benchmark models has been depicted in Figure 7. This figure provides a balanced perspective on performance versus computational complexity.

**Table 6:** Ablation study assessing the incremental impact of SAEB and DAEB encoders and SegPath on segmentation metrics (DSC, IOU, AHD) across BRATS 2021, ATLAS R2.0, and ISLES 2022 datasets.

Network	DSC	IoU	AHD
Brats 2021 dataset			
Baseline (U-Net)	0.86	0.76	7.01
Baseline + SegPath	0.87	0.76	6.99
Baseline + SAEB	0.87	0.78	6.65
Baseline + SAEB + SegPath	0.89	0.80	6.26
Baseline + DAEB	0.88	0.80	6.35
Baseline + DAEB + SegPath	0.90	0.81	6.07
ATLAS R2.0 Dataset			
Baseline (U-Net)	0.72	0.56	11.8
Baseline + SegPath	0.73	0.57	11.6
Baseline + SAEB	0.75	0.60	11.2
Baseline + SAEB + SegPath	0.77	0.62	10.6
Baseline + DAEB	0.76	0.61	11.0
Baseline + DAEB + SegPath	0.78	0.63	10.4
ISLES 2022 Dataset			
Baseline (U-Net)	0.76	0.61	11.51
Baseline + SegPath	0.77	0.61	11.23
Baseline + SAEB	0.79	0.66	10.24
Baseline + SAEB + SegPath	0.81	0.68	9.855
Baseline + DAEB	0.79	0.65	10.25
Baseline + DAEB + SegPath	0.80	0.67	10.04

In summary, our ablation studies, built on the baseline 3D U-Net model, attest to the efficacy of our proposed encoders. The summarized results and conclusions can be found in Table 6. By offering these two encoder alternatives, we allow users to choose between SAEB’s computational efficiency or DAEB’s slightly superior performance, depending on their specific requirements.

#### 5.2.1 Ablation study for SAEB

We have conducted a comprehensive ablation study to evaluate the impact of integrating the Single Adaptive Encoder Block (SAEB) with the SegPath module. As seen in the results presented in Table 6, the fusion of SAEB with SegPath, as summarized by the AFMS-SAEB configuration, demonstrates substantial improvements across all examined datasets. Reviewing the BraTS 2021 dataset shows a marked enhancement in DSC and IoU metrics by integrating the SAEB and SegPath modules. In particular, the IoU increased from 76.7 to 81.6%, and the DSC score increased from the starting value of 86.7 to 89.4%. Same for ISLES 2022 and ATLAS R2.0 datasets. The outcomes show that the AFMS-SAEB model can accurately represent the edges of lesions and other small features, which are critical for medical image segmentation. The AFMS-SAEB’s precise ability results from the SAEB module’s feature extraction power and SegPath’s capability in contextual capture, which precisely detects intricate anatomical and clinical characteristics. To sum up, Table 6 presents compelling evidence about the efficacy of SAEB and SegPath’s combined competence inside the AFMS-SAEB model. Our ablation research demonstrates that AFMS-SAEB has considerable efficiency in fine-grain identification and segmentation and enhances the accuracy of image segmentation.

#### 5.2.2 Ablation study for DAEB

The AFMS-DAEB is designed for the Dual-Dimension Attention mechanism purpose by the strategic integration of DAEB, SegPath, and decoder module (Table 6), demonstrates the performance and robustness of AFMS-DAEB for complex anatomical and pathological structures across various medical imaging datasets. For the Brats dataset, the proposed AFMS-DAEB significantly improved over the baseline method in DSC and IoU, from 86 to 90% and 76 to 81%, respectively. Due to the dual attention mechanism, the DAEB module can detect subtle lesions that most models may overlook. Improvements in the ATLAS R2.0 and ISLES 2022 datasets further validate the model’s efficacy. The AFMS-DAEB emphasizes the importance of extracting details-oriented features. DAEB and SegPath modules, ensures that the model preserves and maintains a holistic understanding of a spatial context while extracting finer details, edges, and complex contrasts. Because of the DAEB’s robustness, the model can extract the most contextual information from medical images, which helps it overcome the difficulties presented by subtle variances in medical imaging. In the meantime, the SegPath improves this by supporting the processing and hierarchical structuring of the learned features.

#### 5.2.3 Ablation study for SegPath

To evaluate the effectiveness of SegPaths, we integrate the SegPaths with the base model U-Net to conduct quantitative analysis. The results are shown in Table 6. All three datasets had an improvement in DSC scores; BraTS, ATLAS, and ISLES registered scores of 87.1, 73.6, and 77.1%, respectively. In the Baseline + SAEB versus Baseline + SAEB + SegPath (AFMS-SAEB) comparison, the combination of SAEB and SegPath performed better. The DSC score increased from 88.7 to 89.4%, and the IoU score increased from 80.8 to 81.6% for the BraTS 2021. Notable improvements were also observed in the ATLAS and ISLES 2022 datasets, demonstrating the cooperative effect of the SAEB and SegPath. The model with Baseline + DAEB + SegPath (AFMS-SAEB) showed remarkable results at the end of our investigation, particularly when compared to the Baseline + DAEB. For example, the BraTS 2021 outperformed all previous architectures with DSC and IoU ratings of 90.2 and 82.3%, respectively. In Summary, SegPath dramatically improves the model’s capacity for feature refinement.

## 6 Conclusion and future work

Deep learning models must effectively capture local and global features to perform accurate and efficient brain lesion segmentation. Previously, many state-of-the-art methods such as U-Net, VGG-Net, ResNet, and DenseNet have set the foundation. However, these methods may fail in precisely segmenting brain lesions due to the brain’s complex structure. Moreover, these methods could face computational overload. Thus, we introduce a novel network AFMS-Net to optimize segmentation accuracy and computational efficiency. Our proposed network has an encoder-decoder-like architecture that includes SAEB and DAEB modules. These encoder structures represent a notable shift in feature extraction, enhanced by techniques such as squeeze-and-excite and channel-spatial attention. The SAEB and DAEB utilized SegPath by combining residual and traditional skip connections for adaptive feature accumulation, which is further responsible for capturing and enhancing detailed features and multi-scale context for improved segmentation outcomes. Thus, it is suitable for limited computational resources, or the primary target is identifying and segmenting the most prominent features. SAEB is ideal for fast and efficient segmentation in scenarios prioritizing speed, unsuitable for complex, detailed analysis. DAEB excels in precise, intricate segmentation tasks, especially with multi-class lesions, not recommended for rapid, less detailed screenings.

The experimental findings of the AFMS-SAEB module demonstrated impressive performance in terms of Dice and IoU scores. For the BraTS dataset, 89.4% of Dice and 80.6% of IoU scores were achieved. The ATLAS scores were recorded as 77.2 and 62.4%, while on the ISLES dataset, the Dice and IoU scores were 81.8 and 68.0%, respectively. Compared to other models, it achieved a 2.7% improvement in Dice and 3.9% in IoU compared to U-Net, surpassing Attention U-Net by 3.4 and 4.8%, ResUNet++ by 2.5 and 3.2%, Multi-ResUNet by 3.8 and 5.4%, CS2-Net by 3.9 and 5.8%, and ER-Net by 3.3 and 4.5% on BRATS. Conversely, the proposed AFMS-DAEB module is suitable for fine-grained and complex segmentation tasks that utilize GAP, channel spatial, and weighted channel attention. It emphasizes information channels and integrates spatial attention to identify and classify various lesion types. AFMS-DAEB’s effectiveness is validated through rigorous experiments on several datasets. On BraTS, it achieved remarkable Dice and IoU scores of 90.2% and 0.81.3%, respectively, showcasing its capability in handling complex brain tumor segmentation tasks. For ATLAS and ISLES, it achieved 78.2 and 80.2% (Dice scores) and 63.6 and 67.3% (IoU scores), supporting the model’s robustness and versatility across different medical imaging challenges. Results across all datasets show that AFMS-DAEB performs better than the baseline U-Net model. Regarding Dice and IoU, it improved by 3.5 and 4.6% on BraTS, respectively. Performances were considerably greater on ATLAS, with an increase of 7% in IoU and 6.1% in Dice. The model demonstrated outstanding results: a rise of 5.9% in IoU and 4.1% in Dice on the ISLES dataset.

Furthermore, our study has some limitations because it only used high-resolution MRI scans, which may not accurately reflect the range of clinical circumstances that are seen in real-world settings. To be more specific, the performance of the AFMS-Net on datasets such as BraTS 2021, ATLAS v2.0, and ISLES 2022, which have voxel sizes of 1 × 1 × 1 mm and 2 × 2 × 2 mm respectively, demonstrates its ability in high-resolution context setting. When applied to lower-resolution images or other imaging modalities, which are often used in a variety of diagnostic contexts, this approach may raise concerns regarding the model’s efficacy and flexibility. This limitation highlights the possibility of bias in the model towards the high-resolution features included in the datasets that were utilized, and it may raise the possibility of a compromise in the generalizability of the model. In order to overcome these issues, future research will focus on AFMS-Net’s usefulness across various imaging modalities in addition to evaluating and improving its ability to adapt to images of various resolutions.

We will also refine our approach to parameter tuning and explore the potential of leveraging unsupervised learning for 3D medical image segmentation. In our forthcoming work, we aim to expand interdisciplinary collaborations that will augment the clinical applicability of our models. Through these collaborative efforts, we anticipate that AFMS-Net will profoundly influence clinical decision-making by facilitating precise and efficient lesion segmentation. In conclusion, AFMS-Net represents a significant advancement in medical image segmentation.

## Data availability statement

The datasets used in this study can be found in online repositories, and the names of the repository/repositories and accession number(s) are provided in the article.

## Author contributions

AZ: Methodology, Software, Validation, Visualization, Writing – original draft, Writing – review & editing, Conceptualization, Data curation, Formal analysis, Investigation. HH: Data curation, Investigation, Writing – review & editing. XZ: Data curation, Investigation, Software, Writing – review & editing. RK: Formal analysis, Validation, Visualization, Writing – review & editing. JL: Data curation, Software, Validation, Writing – review & editing. HY: Data curation, Formal analysis, Software, Writing – review & editing. XM: Data curation, Investigation, Software, Writing – review & editing. AC: Investigation, Software, Validation, Writing – review & editing. YY: Data curation, Formal analysis, Investigation, Software, Writing – review & editing. BH: Investigation, Software, Validation, Writing – review & editing. YG: Formal analysis, Investigation, Methodology, Software, Writing – review & editing, Validation, Visualization. YK: Conceptualization, Data curation, Formal analysis, Funding acquisition, Investigation, Methodology, Project administration, Resources, Software, Supervision, Validation, Visualization, Writing – review & editing.
